# Estimating malaria parasite density among pregnant women at central Sudan using actual and assumed white blood cell count

**DOI:** 10.1186/1475-2875-13-6

**Published:** 2014-01-05

**Authors:** AbdElrahium D Haggaz, Leana M Elbashir, Gamal K Adam, Duria A Rayis, Ishag Adam

**Affiliations:** 1 Faculty of Medicine, University of Geizera, PO Box 412, Medani, Sudan; 2 Faculty of Medicine, University of Khartoum, PO Box 102, Khartoum, Sudan; 3 Faculty of Medicine, University of Gadarif, Gadarif, Sudan

**Keywords:** Malaria, Pregnancy, Diagnosis, Microscopy, Sudan

## Abstract

**Background:**

Microscopic examination using Giemsa-stained thick blood films remains the reference standard for detection of malaria parasites and it is the only method that is widely and practically available for quantifying malaria parasite density. There are few published data (there was no study during pregnancy) investigating the parasite density (ratio of counted parasites within a given number of microscopic fields against counted white blood cells (WBCs) using actual number of WBCs.

**Methods:**

Parasitaemia was estimated using assumed WBCs (8,000), which was compared to parasitaemia calculated based on each woman’s WBCs in 98 pregnant women with uncomplicated *Plasmodium falciparum* malaria at Medani Maternity Hospital, Central Sudan.

**Results:**

The geometric mean (SD) of the parasite count was 12,014.6 (9,766.5) and 7,870.8 (19,168.8) ring trophozoites /μl, P <0.001 using the actual and assumed (8,000) WBC count, respectively. The median (range) of the ratio between the two parasitaemias (using assumed/actual WBCs) was 1.5 (0.6-5), i e, parasitaemia calculated assuming WBCs equal to median (range) 1.5 (0.6-5) times higher than parasitaemia calculated using actual WBCs. There were 52 out of 98 patients (53%) with ratio between 0.5 and 1.5. For 21 patients (21%) this ratio was higher than 2, and for five patients (5%) it was higher than 3.

**Conclusion:**

The estimated parasite density using actual WBC counts was significantly lower than the parasite density estimated using assumed WBC counts. Therefore, it is recommended to use the patient`s actual WBC count in the estimation of the parasite density.

## Background

The World Health Organization (WHO) recommends a parasite-based diagnosis of malaria infection [1]. Microscopic examination using Giemsa-stained blood slides for detection of malaria parasites remains, in spite of the availability of the other various methods, the reference standard and it is routinely relied upon as a primary endpoint measurement for patient care, epidemiological studies, operational research, intervention studies, as well as clinical trials [1,2]. It is the only method that is widely and practically available for quantifying malaria parasite density by comparing the ratio of counted parasites within a given number of microscopic fields, against either counted white blood cells (WBCs) or counted red blood cells (RBCs) within those same fields, and then multiplying that ratio by either the measured or estimated/assumed density of the patient’s actual WBCs or RBCs [1,2].

Due to the lack of laboratory facilities in most malaria-endemic countries to quantify patients’ WBCs, an assumed WBC count of 8.0 × 10^9^/L, which is set by WHO, is currently/conventionally the practice to estimate malaria parasite densities [3]. Yet, using the conventional method of assumed WBCs of 8.0 × 10^9^/L to quantify parasite densities may generate errors which could influence conclusions [4,5]. Thus, the use of assumed WBCs rather than actual WBCs might lead to over-estimation or under estimation of the parasite density in the case of other infections, or the contrary, depending on several factors, such as severity of malaria and other infections and the acceptable reference value for WBCs in the specific settings [5,6]. Furthermore, WBC count, particularly in malaria-infected individuals, can vary by as much as ten-fold between individuals [4]. Previously, WBC count was a technically challenging procedure that needed human expertise, but now full blood counts (particularly WBC count) can be performed with automated haematology analyzers. There are few published studies investigating parasite density using actual WBC counts rather than assumed ones [6-8], and no studies have been conducted among pregnant women. Thus, the current study was conducted to investigate the parasite density among pregnant women using actual WBC counts so as to improve the previous diagnostic methods for malaria during pregnancy in Sudan [9-12]. Malaria during pregnancy is a major health problem where pregnant Sudanese women are at risk of malaria regardless of their age or parity and malaria is associated with adverse maternal and perinatal outcomes [13].

## Methods

A cross-sectional study was conducted at Medani Maternity Hospital in Central Sudan from August to December 2011. The area is characterized by unstable malaria transmission and *Plasmodium falciparum* is the sole malaria species in the area [14]. After signing an informed consent, febrile (temperature ≥37.5°C) pregnant women who attended the antenatal clinic were screened and clinically examined for malaria. Information on social demographics was collected using a pre-tested questionnaire. Malaria screening was performed using microscopy. Pregnant women with a confirmed diagnosis of malaria were treated with artesunate sulphadoxine-pyrimethamine if they were in the second and third trimesters and by quinine if they were in the first trimester [15,16]. The sample sizes were calculated based on two-sided hypothesis tests using Epi Info with 80% power and a confidence interval of 95%.

### Microscopy

Thick blood smears (each slide had a measured volume of 6 μl of blood) were stained with 10% Giemsa and examined under the ×100 oil immersion objective lens of a light microscope by two independent laboratory technicians who were blinded to each other's results. The number of asexual parasites was counted against 200 leucocytes, where an average leucocytes count of 8,000/μL was assumed. Before a smear was considered negative, 200 high power fields had been examined.

### Estimation of parasite density

Parasite densities were recorded as a ratio of parasites to WBCs in thick films. Plasmodium parasites were counted against 200 WBC on the thick film. Five hundred WBCs were counted where less than nine parasites were counted after counting against 200 WBC. Parasite densities (parasite/μl of whole blood) were then calculated as follows:

$$\begin{array}{l} =\left(\mathrm{Number}\;\mathrm{of}\;\mathrm{parasites}\;\mathrm{counted}/\mathrm{WBC}\;\mathrm{counted}\right)\\ \;\times \mathrm{WBC}\;\mathrm{count}/\mathrm{\mu L}\;\mathrm{of}\;\mathrm{participant}. \end{array}$$

Also, parasite densities for all participants were calculated using assumed WBC counts of 8.0 × 10^9^/L of blood; all set by WHO (as mentioned above) to be used conveniently in facilities which lack the tools to determine patients’ absolute full blood count value [3].

### Automated haemoanalyzer

The details of the haemoanalyzer were provided in the previous recent work [17]. In summary, 2 ml of blood was taken in an ethylene diamine tetra acetic acid and immediately analysed for a complete haemogram, including WBC count, using an automated haematology analyzer. The Sysmex KX21N is an automated blood cell counter used for *in vitro* diagnostic in clinical laboratories. It is a compact, fully automated haematology analyzer with simultaneous analysis of 18 parameters in whole blood mode and capillary blood mode. This analyzer counts blood cells as routine in a few minutes. The test was performed as stated in the manufacturer’s instructions [18].

### Ethics

The study received ethical clearance from the Research Board at the Faculty of Medical Laboratory Sciences, University of Khartoum, Sudan.

### Statistics

The results were analysed using SPSS, version 16.0 for Windows (SPSS Inc, Chicago, IL, USA). Non-parametric tests were used to compare the geometric mean of the parasite count. There was no randomness in the difference between the two measurements of parasite density, where the same count of parasites (which was a random variable) was used for both methods (actual and assumed WBC count): the parasite density using the assumed WBCs (8,000)/number of cells counted and the actual/number of cells counted. Therefore, the ratio of the two parasitaemias was: parasite density using assumed WBCs/parasite density using the actual WBC count = 8,000/actual WBC count, i e, determined by WBCs. For a given patient with a determined white cell count, the ratio of parasitaemias calculated in these two ways will be constant, irrespectively of the number of parasites counted.

## Results

**Figure 1:**
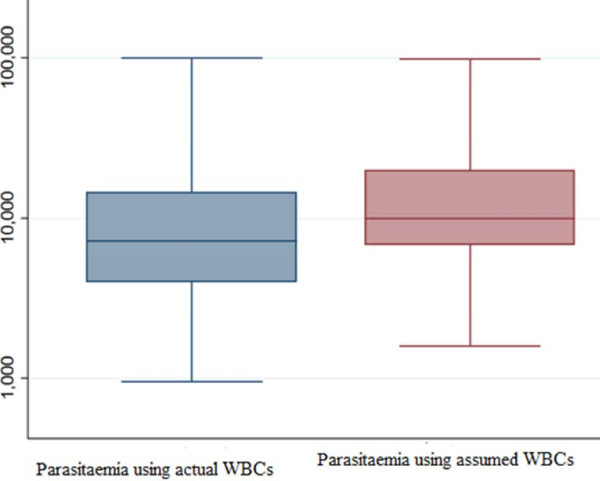
Comparing parasitaemia using assumed and actual white blood cell counts.

**Figure 2:**
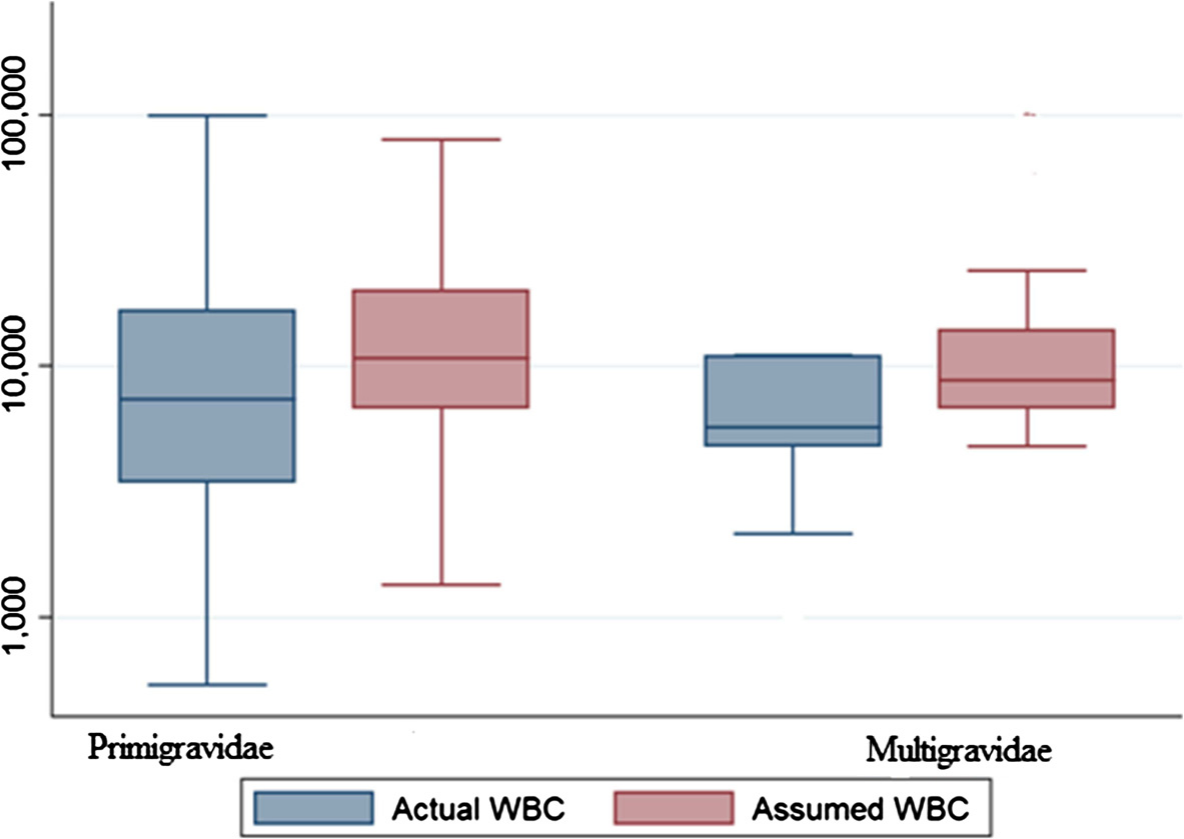
**Comparing parasitaemia in primigravidae and multigravidae using assumed and actual white blood cell c** ***ounts.***

The basic characteristics of the enrolled women are shown in Table 1. The number of WBCs ranged from 1,600 to 14,000 while the median was 5,500 cells/μ. The geometric mean (SD) of the parasite count was 12,014.6 (9,766.5) and 7,870.8 (19,168.8) ring trophozoites/μl, P <0.001 using the actual and assumed (8,000) WBC count, respectively (Figure 1). Likewise, in primigravidae the geometric mean (SD) of the parasite count was 11,114.1 (23,924.9) and 8,199.3 (25,771.7), P <0.001, using actual and assumed WBC count, respectively (Figure 2).

**Table 1:** Participant demographics

**Variables**	**Mean (SD) [range]**
Age, years	27 (5.8) [17–40]
Parity	2.8 (2.3) [0–9]
Gestational age, week	26.5 (8.7) [6–38)
Haemoglobin, g/dl	9.3 (1.9) [5-12]
White blood cells/μ	*5,500 [1,600-14,000]

*median.

**Figure 3:**
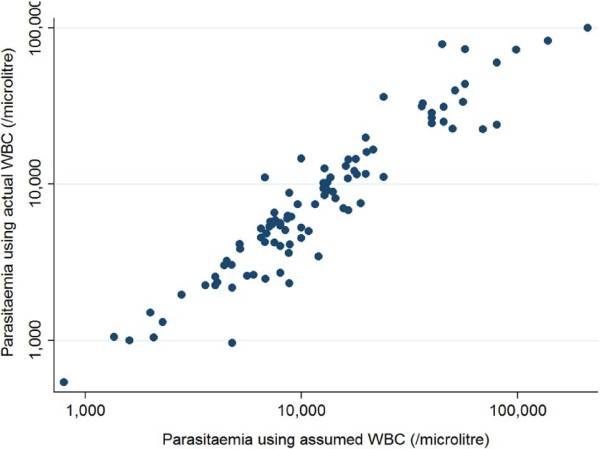
Correlations between parasitaemia using assumed and actual white blood cell counts.

**Figure 4:**
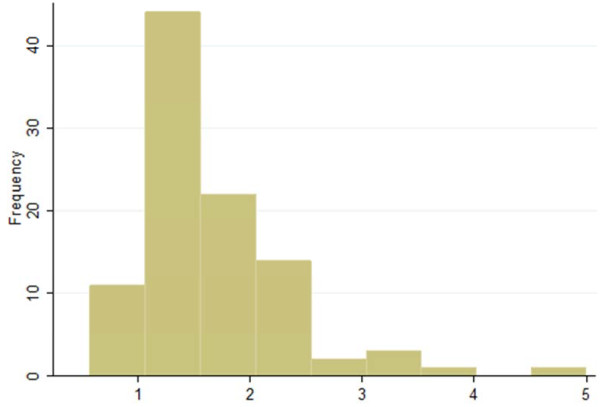
Ratios of parasitaemias that were assessed by assumed and actual white blood cell counts.

The median (range) of the ratio between the two parasitaemias was 1. 5 (0.6 -5), i e, parasitaemia calculated assumed WBCs (8,000) was equal to median (range) 1.5 (0.6-5) times higher than parasitaemia calculated using actual WBC counts. There were 52 out of 98 patients (53%) with ratio between 0.5 and 1.5. For 21 patients (21%) this ratio was higher than 2, and for five patients (5%) it was higher than 3 (Figures 3 and 4).

## Discussion

The main finding of the current study was that the parasite density, which was calculated using the patients` own WBC counts, was significantly lower than that calculated using the assumed WBC counts (8,000). It was previously proposed that the best solution in estimating parasite densities (for children) would be to use the corresponding standard WBC counts for each age group [19]. The results of the current study are in agreement with the findings of Jeremiah and Uko, who conducted a cross-sectional study in Nigerian children and found that the assumed parasite counts with the standard method overestimated parasitaemia, resulted from the patients` actual WBC counts [6]. Recently, in Central Ghana Adu-Gyasi *et al*. [7] observed that the assumed WBC of 8.0 × 10^9^/L or lower resulted in significant underestimation of parasite density of *P. falciparum* malaria parasite in 5,902 malaria microscopy positive slides in groups of children less than 5 years old. Yet, they found no significant difference in geometric mean parasite density with assumed WBCs of 10.0 × 10^9^/L [7]. It is worth to be mentioned that the low haemoglobin level (mean = 9.3 g/dl) and the median number WBCs of 5500 cells/μl would have influenced the results of parasite density and these have to be taken in consideration when comparing the results of the current study with the findings of others.

The current study and the above mentioned studies did not support the use of assumed (8,000) WBC counts in estimating the parasite density as recommended by WHO [3].

## Conclusion

The estimated parasite density using actual WBC counts was significantly lower than the density estimated using assumed (8,000) WBCs in pregnant women at central Sudan. It is, therefore, recommended that actual WBCs are used in the estimation of parasite densities.

## Competing interests

The authors declare that they have no competing interests.

## Authors’ contributions

ADH and IA coordinated and carried out the study. IA, GKA and DAR participated in the statistical analysis. LME and GKA participated in the clinical work and conducted the laboratory work. All the authors have read and approved the final version of this manuscript.
